# Retinoid-Induced Expression and Activity of an Immediate Early Tumor Suppressor Gene in Vascular Smooth Muscle Cells

**DOI:** 10.1371/journal.pone.0018538

**Published:** 2011-04-05

**Authors:** Jeffrey W. Streb, Xiaochun Long, Ting-Hein Lee, Qiang Sun, Chad M. Kitchen, Mary A. Georger, Orazio J. Slivano, William S. Blaner, Daniel W. Carr, Irwin H. Gelman, Joseph M. Miano

**Affiliations:** 1 Aab Cardiovascular Research Institute, University of Rochester School of Medicine and Dentistry, Rochester, New York, United States of America; 2 Department of Pathology and Laboratory Medicine, University of Rochester School of Medicine and Dentistry, Rochester, New York, United States of America; 3 Department of Medicine, Columbia University, New York, New York, United States of America; 4 Portland Veterans Affairs Medical Center, Portland, Oregon, United States of America; 5 Department of Cancer Genetics, Roswell Park Cancer Institute, Buffalo, New York, United States of America; University of Padova Medical School, Italy

## Abstract

Retinoids are used clinically to treat a number of hyper-proliferative disorders and have been shown in experimental animals to attenuate vascular occlusive diseases, presumably through nuclear receptors bound to retinoic acid response elements (RARE) located in target genes. Here, we show that natural or synthetic retinoids rapidly induce mRNA and protein expression of a specific isoform of A-Kinase Anchoring Protein 12 (AKAP12β) in cultured smooth muscle cells (SMC) as well as the intact vessel wall. Expression kinetics and actinomycin D studies indicate *Akap12β* is a retinoid-induced, immediate-early gene. *Akap12β* promoter analyses reveal a conserved RARE mildly induced with atRA in a region that exhibits hyper-acetylation. Immunofluorescence microscopy and protein kinase A (PKA) regulatory subunit overlay assays in SMC suggest a physical association between AKAP12β and PKA following retinoid treatment. Consistent with its designation as a tumor suppressor, inducible expression of AKAP12β attenuates SMC growth in vitro. Further, immunohistochemistry studies establish marked decreases in AKAP12 expression in experimentally-injured vessels of mice as well as atheromatous lesions in humans. Collectively, these results demonstrate a novel role for retinoids in the induction of an AKAP tumor suppressor that blocks vascular SMC growth thus providing new molecular insight into how retiniods may exert their anti-proliferative effects in the injured vessel wall.

## Introduction

Vascular SMC are normally quiescent and express a repertoire of cytoskeletal and contractile proteins that subserve functions related to contractile tone and the maintenance of vascular integrity. A variety of vasculopathies shift the phenotype of SMC from one of quiescence and contractile competence to proliferation, migration, matrix production, and attenuated expression of contractile proteins [1]. A variety of therapeutic molecules have been shown to attenuate such phenotypic switching including a class of compounds known as retinoids [2]. Retinoids encompass synthetic and natural derivatives of retinol (vitamin A) that have found clinical utility in the management of several human hyper-proliferative disorders [3], [4]. Cultured SMC treated with the natural retinoid, all-trans retinoic acid (atRA) or its isoform (*e.g.*, 9-cis RA), consistently show reduced growth potential during growth factor stimulation [5]–[9] and in some cases restoration of the contractile phenotype [10]. Moreover, a variety of animal models of vascular disease have been used to demonstrate retinoid-mediated decreases in neointimal burden and increases in vessel patency [11]–[19]. Thus, retinoids represent a viable class of therapeutic molecules for the potential management of vascular occlusive disorders.

Retinoids exert their pleiotropic actions by binding ligand-activated nuclear receptors that control gene expression [20]. One logical approach to begin elucidating the mechanisms underlying retinoid action in the vessel wall is to define retinoid-responsive target genes. We previously performed a modified suppression subtractive hybridization screen in cultured SMC for the identification of atRA-responsive genes [21]. One of the genes reported to be induced by atRA was Src-Suppressed C Kinase Substrate (SSeCKS), the rodent ortholog [22] of human gravin [23] encoding for an A-kinase anchoring protein (AKAP). SSeCKS (official gene symbol, AKAP12) binds and localizes a number of signaling proteins including PKA, protein kinase C (PKC), calmodulin, and the β_2_-adrenergic receptor [24], [25]. The assembly of such signaling complexes is linked to AKAP12 activities involved in growth suppression, actin cytoskeletal remodeling, and adrenergic signal transduction [24]. The growth suppressive activities of AKAP12, together with its attenuated expression in both transformed cell lines and a variety of human neoplasms, have led to the concept of AKAP12 being a tumor suppressor gene [24]. Further evidence for this hypothesis was recently demonstrated in AKAP12 knockout mice, which exhibit prostatic hyperplasia and focal dysplasia [26].

Previous studies have documented AKAP12 expression in SMC [21], [27], [28], but the regulatory control of its induction and function in SMC have not been well characterized. Moreover, since the AKAP12 locus comprises three independent transcription units, each under control of a unique promoter [29], the AKAP12 isoform responsive to the action of retinoids is unknown. In this report, we show that the AKAP12β isoform is rapidly and highly induced by both natural and synthetic retinoids. We further show that AKAP12β associates with PKA and mediates increases in activity of at least one downstream target of PKA. Acute or inducible over-expression of AKAP12β attenuates SMC growth in human and rodent SMC model systems. Finally, we demonstrate decreases in AKAP12 expression in vascular lesions where hyper-proliferative activity exists. Our results establish AKAP12β as a novel retinoid-responsive tumor suppressor gene, making it an attractive target for therapy in a variety of disease contexts, including vascular occlusive diseases.

## Results

### atRA Induces a Specific Isoform of AKAP12 in Multiple Species of Vascular SMC

**Figure 1:**
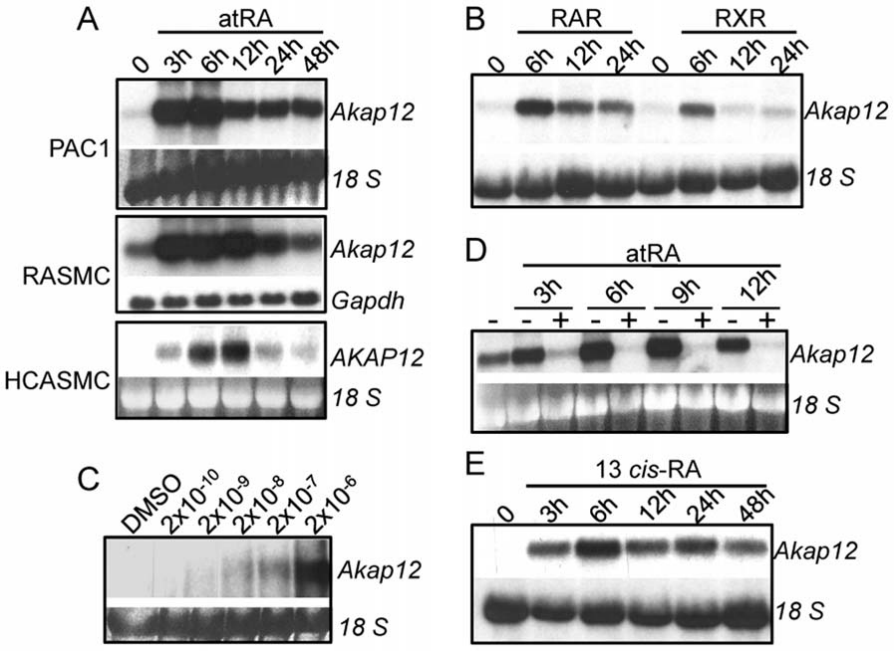
Retinoid-induced expression of *Akap12*. Northern blot analyses of *Akap12* expression showing (A) time-dependent increases following atRA stimulation in the indicated cells; (B) retinoid receptor agonist-induced *Akap12* mRNA in PAC1 SMC; (C) atRA dose-dependent increase in PAC1 SMC; and (D) RNA polymerase II-dependent increase in PAC1 SMC treated with atRA in absence or presence of actinomycin D (Act-D). Equivalent total RNA loading is indicated with either ethidium bromide staining of 18 S rRNA or expression levels of glyceraldehyde phosphate dehydrogenase (*Gapdh*).

In the course of defining a novel retinoid-response gene set, we identified a tumor suppressor gene called AKAP12 that was induced with atRA in RASMC [21]. To extend these results, we performed Northern blotting on multiple sources of SMC treated for varying times with atRA. Results in Figure 1A reveal a rapid induction of *Akap12* mRNA in the rat PAC1 SMC line, RASMC, and HCASMC. The increase in *Akap12* mRNA was also seen with 13 *cis*-RA stimulation, but not with agonists to PPAR gamma (data not shown). On the other hand, agonists to retinoic acid receptor (RAR) and retinoid X receptor (RXR) each elicited increases in *Akap12* mRNA (Figure 1B). The increase in *Akap12* mRNA with atRA was dose-dependent (Figure 1C) and RNA polymerase II-dependent as evidenced by complete suppression with actinomycin D treatment (Figure 1D). atRA-stimulated *Akap12* mRNA was not universally seen as some cell types (L6 and BC_3_H1 myoblasts) failed to show increases with retinoid treatment (data not shown). *Akap12*'s rapid expression kinetics in SMC following retinoid stimulation, its dependence on de novo mRNA synthesis, and its independence for de novo protein synthesis [21] indicate that this tumor suppressor is a retinoid-induced, immediate-early gene.

**Figure 2:**
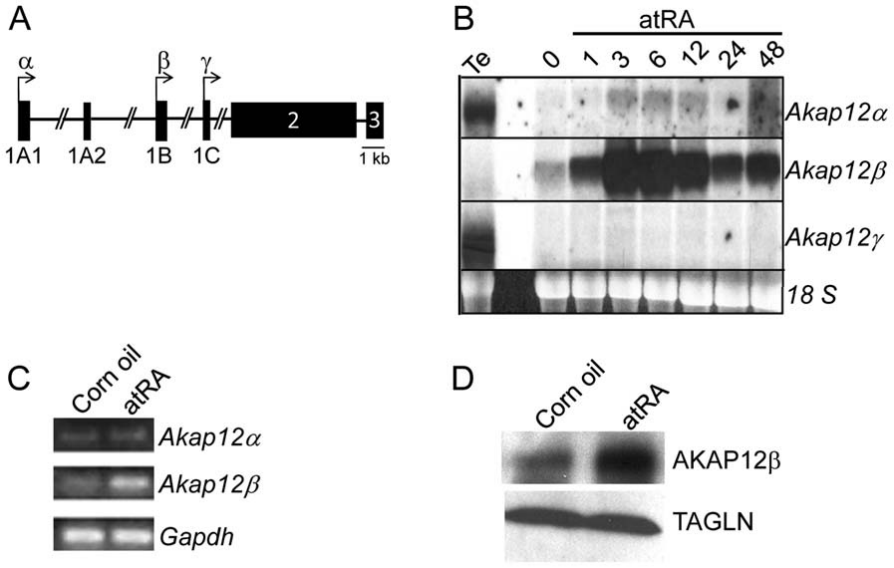
atRA-induced expression of the *Akap12β* isoform. (A) Schematic of *Akap12* locus comprising three alternate start sites of transcription (bent arrows) under separate promoter control with proper nomenclature for exons. (B) Northern blotting of PAC1 SMC treated with atRA for the indicated times and using exon-specific probes to each *Akap12* isoform. Testes RNA (Te) is included as a positive control for the *Akap12α* and *Akap12γ* isoforms. (C) Northern blotting with isoform-specific PCR primers on aortic tissue from mice treated with either corn oil or atRA for 24 hr. (D) Western blotting with antisera to AKAP12 on aortic tissue from mice treated with either corn oil or atRA for 24 hr suggests a similar elevation of AKAP12β protein (see also Figure 4A).

We recently defined the *Akap12* genomic landscape and discovered that three independent promoters direct expression of three *Akap12* isoforms (Figure 2A and [29]). To ascertain which of the *Akap12* isoforms is targeted for induction with retinoids, exon-specific probes to each *Akap12* isoform were designed and applied to samples of RNA from atRA-stimulated PAC1 SMC. Results indicate that atRA specifically targets the *Akap12β* isoform with expression kinetics nearly identical to those observed with a probe common to all *Akap12* isoforms (compare Figure 1A with Figure 2B). Similar kinetics of *Akap12β* induction was seen with the synthetic retinoid AM80 (Figure S1A). mRNA kinetic studies suggest that the half-life of retinoid-induced *Akap12β* is on the order of 3 hr (Figure S1B).

To determine whether retinoids elicit *Akap12β*-specific induction in the intact vessel wall, we administered atRA or corn oil to adult mice by oral gavage and measured serum retinoid levels as well as *Akap12* isoforms in vascular tissue (enriched for SMC only) using PCR primers specific for *Akap12α* or *Akap12β.* No detectable levels of retinoids were seen in corn oil treated mice. However, consistent with a previous report in the rat [11], atRA-treated mice exhibited therapeutic levels of atRA (5144.8±701 ng/ml), 13-cis RA (410.9±86 ng/ml) and 9-cis RA (29.7±9 ng/ml) 6 hr following atRA administration. As with in vitro cell culture data, SMC- enriched vascular tissue exposed to atRA showed little change in *Akap12α* mRNA but clear increases in the *Akap12β* isoform (Figure 2C). A similar induction of AKAP12 protein was observed in vascular SMC-enriched aortic tissue (Figure 2D). Taken together, results indicate rapid and specific induction of the *Akap12β* isoform upon treatment with retinoids in various SMC culture models as well as mouse SMC of the vessel wall.

### *Akap12β* Promoter Harbors an Atypical RARE

**Figure 3:**
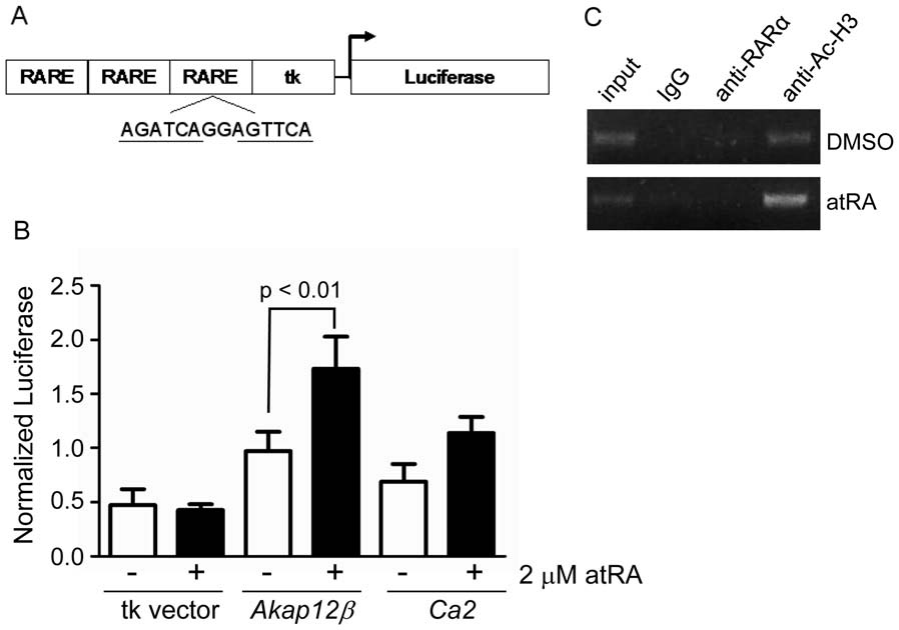
*Akap12β* RARE transcriptional activity and chromatin remodeling. (A) Schematic of multimerized RARE (located on antisense stand -2.5 kb upstream of *Akap12β* promoter; see coordinates 2866-2879 in GenBank Accession number AY695060) cloned upstream of a thymidine kinase (tk) luciferase expression plasmid. The two half-sites of the RARE are underlined. (B) PAC1 SMC transfected with luciferase reporter plasmids containing indicated multimerized RARE sequences in absence or presence of atRA. Data are normalized to the Renilla control plasmid and represent the average of three replicates. Data are representative of several similar transient transfections in PAC1 SMC. (C) ChIP assay of PAC1 SMC treated with DMSO or atRA for 6 hr using the indicated antibodies. Similar enrichment of acetylated histone H4 was seen with atRA stimulation (data not shown).

We previously characterized the *Akap12β* promoter and showed basal activity in a variety of cell types, including SMC [29]. To determine whether any conserved RARE is present in the *Akap12β* promoter, we compared the rat, mouse, and human *Akap12β* promoters for conserved RAREs based on a base frequency table of 67 experimentally-validated RAREs [4]. We found an RARE located -2,534 bp upstream of the annotated start site of transcription (see GenBank Accession number AY695060) and the sequence of this RARE indicates that it is a direct repeat (DR)-2 RARE, where each half site is spaced by two nucleotides (Figure 3A). Extensive transient and stable transfections in SMC treated with atRA revealed weak activation of the native *Akap12β* promoter constructs carrying the RARE (data not shown). To determine whether the *Akap12β* RARE is more responsive to atRA in isolation, we multimerized the sequence and placed it upstream of a thymidine kinase minimal promoter (Figure 3A). Robust activation of a DR-5 RARE from the *Rarβ* gene is evident with atRA stimulation (data not shown). However, the *Akap12β* RARE is only weakly (∼2-fold) activated with atRA stimulation (Figure 3B). This level of activation is comparable to that seen with carbonic anhydrase (*Ca2*), a known retinoid responsive target gene containing a similar DR-2 RARE as that seen in the *Akap12β* RARE [30] (Figure 3B). Mutagenesis experiments failed to reveal a clear-cut dependence on the DR-2 RARE (data not shown), likely because of the weak level of induction. Nevertheless, ChIP assays consistently showed enrichment for acetylated histone H3 within the region encompassing the DR-2 RARE, suggesting that the chromatin landscape is modified to a transcriptionally competent state with retinoid stimulation (Figure 3C). Interestingly, we saw little evidence of retinoic acid receptor alpha enrichment in this region consistent with the weak activation of the RARE. These results reveal a mild, though consistent, activation of the *Akap12β* RARE and a transcriptionally competent chromatin landscape following atRA stimulation.

### atRA-Induced AKAP12 Protein Expression and Association with PKA

**Figure 4:**
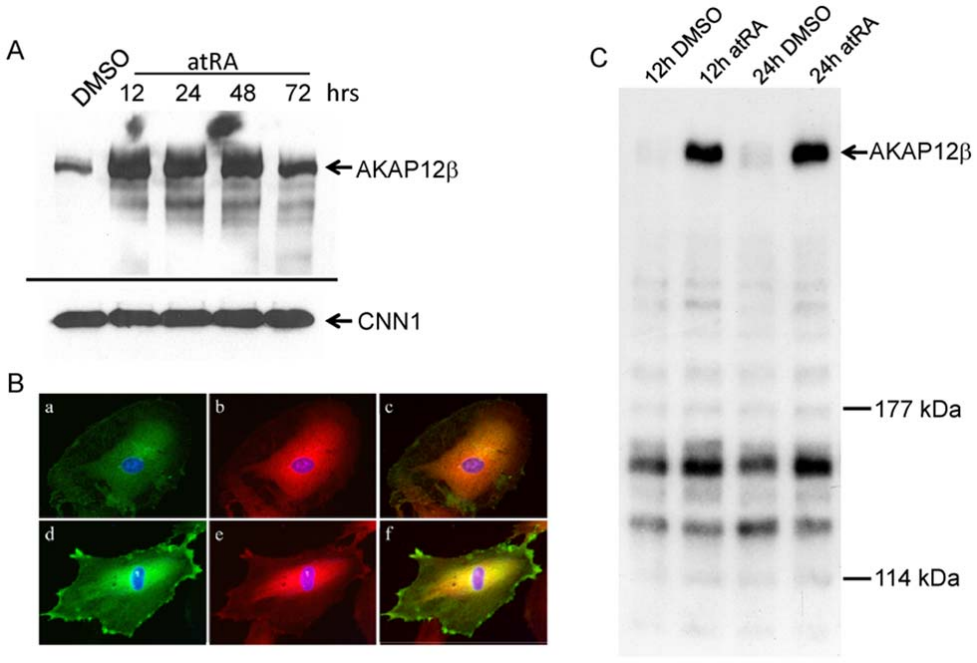
Retinoid-induced AKAP12 protein expression.

To assess expression kinetics of AKAP12 protein, we performed Western blotting on extracts of PAC1 SMC treated with atRA. Results demonstrated increases in AKAP12β protein as early as 12 hr post-atRA treatment with levels gradually decreasing at 72 hr (Figure 4A). Similar induction of AKAP12 protein was seen with AM80 treatment (Figure S2). Virtually no change in expression of smooth muscle calponin (CNN1) was seen with atRA treatment (Figure 4A). Immunofluorescence microscopy of PAC1 SMC showed atRA increases AKAP12 around the nucleus and at the periphery of the cell (Figure 4B, panels a versus d). The expression of the regulatory II (RII) alpha subunit of PKA, to which AKAPs bind [31], displayed a perinuclear localization of expression with little to no changes in level or cellular distribution following atRA stimulation (Figure 4B, panels b versus e, and data not shown). Importantly, the perinuclear expression of AKAP12 and RII alpha appeared to overlap in the perinuclear region only (Figure 4B, panels c versus f). To extend these data further, we performed an RII alpha overlay assay in PAC1 SMC treated with either DMSO vehicle or atRA for 12 and 24 hr. These results demonstrated a dramatic induction of an AKAP12β-radiolabeled RII alpha complex with atRA treatment (Figure 4C). Several lower molecular weight AKAPs showed little, if any, induction suggesting that the stimulatory effect of atRA is specific to the AKAP12 locus in SMC. Interestingly, AKAP12β over-expression studies revealed increases in downstream targets of PKA, namely CREB activation as well as elevated phosphorylation of vasodilator-stimulated phosphoprotein (Figure S3). These results extend our *Akap12β*mRNA expression studies to the protein level and indicate a close association between AKAP12β and PKA-mediated signaling in SMC.

### AKAP12β Overexpression Attenuates SMC Growth

**Figure 5:**
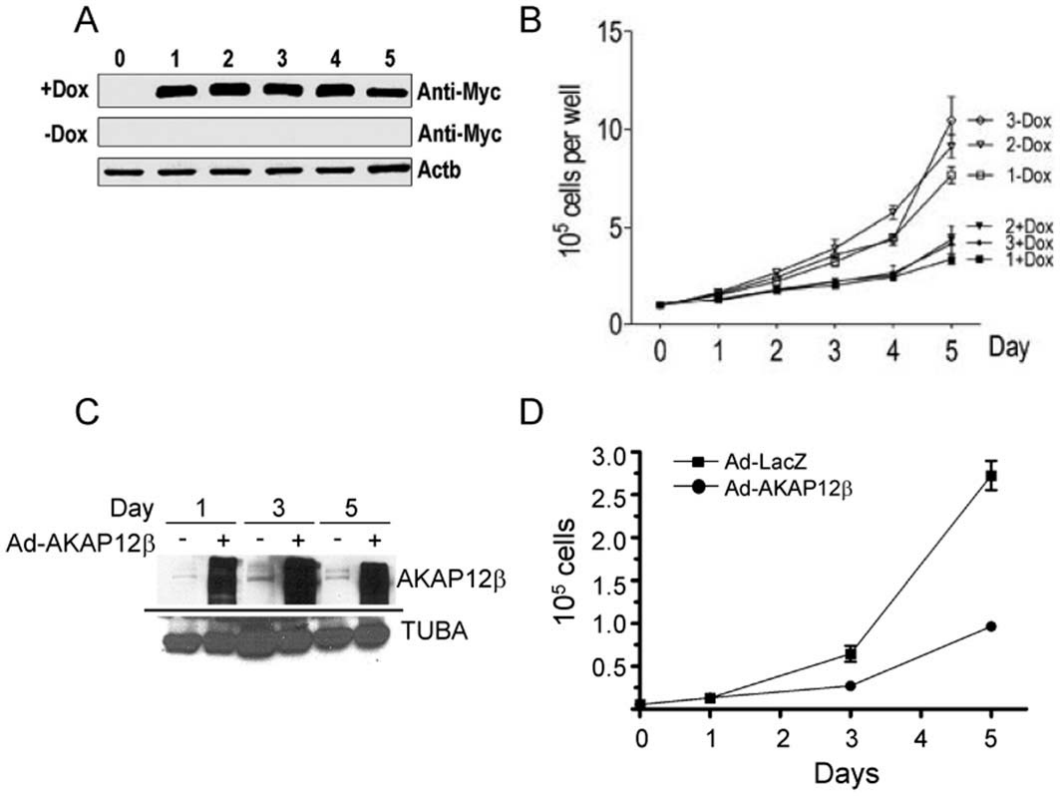
AKAP12β over-expression attenuates SMC growth. (A) Western blot of protein extracts taken from a clone of PAC1 SMC carrying a Myc-tagged AKAP12β transgene stimulated with or without doxycycline (1 µg/ml). Beta actin immunoblot verifies equal protein loading. (B) Parallel dishes of cells carrying Myc-tagged AKAP12β transgene stimulated with or without doxycycline (1 µg/ml) were manually counted with a hemocytometer at the indicated times. Only trypan blue excluding cells were counted. Data are the mean ± SEM of three replicates per time point for each cell line. All three clones carrying AKAP12β showed statistically significant decreases in growth beginning 3 days following Dox stimulation. (C) Western blot showing increases in AKAP12 protein expression within cultured HCASMC transduced with either Ad-AKAP12β (+) or a CMV-driven LacZ adenovirus (−). Blot is representative of two independent experiments. Alpha tubulin immunoblot verifies equal protein loading. (D) Parallel cultures of similarly transduced HCASMC were analyzed for growth over a 5 day period as in panel B. AKAP12β-expressing HCASMC (closed circles) exhibited a statistically significant decrease in growth beginning 3 days following adenoviral transduction as compared to LacZ control cultures (closed squares). Result is representative of two independent experiments performed by different investigators.

Studies in cancer cell lines suggest that both AKAP12α and β attenuate proliferation [24], [32]. To evaluate the potential of AKAP12β over-expression to elicit growth suppressive properties in the context of SMC, we generated PAC1 SMC clones stably-transfected with doxycycline-inducible AKAP12β. Treatment of control (empty vector alone) stable cell lines with doxycycline showed no change in cell growth indicating there was no intrinsic growth inhibitory effects of doxycycline in these cells at a concentration of 1 µg/ml (data not shown). Cells carrying a Myc-tagged AKAP12β transgene showed robust expression of AKAP12β after 1 day of doxycycline treatment with levels AKAP12β persisting over the entire time course of study (Figure 5A). Importantly, we showed doxycycline-dependent cell growth inhibition in three independent AKAP12β expressing cell lines as compared to the same cells where AKAP12β was not over-expressed (Figure 5B). To evaluate the effects of AKAP12β in human SMC, we transduced HCASMC with adenovirus carrying AKAP12β under control of the CMV promoter (Ad-AKAP12β). Efficient over-expression of AKAP12β was shown over a 5 day time course by Western blot analysis (Figure 5C). Similar to results seen in stably-transfected PAC1 SMC, HCASMC over-expressing AKAP12β showed a significant reduction in cell number (Figure 5D). These results are consistent with the known growth suppressive effects of AKAP12 and suggest that retinoids may inhibit SMC growth, at least in part, through the induction of AKAP12β. Attempts to knock down retinoid-induced levels of AKAP12βmRNA were unsuccessful thus precluding rescue studies relating to SMC proliferation (data not shown).

### Expression of AKAP12 in Vascular Lesions

**Figure 6:**
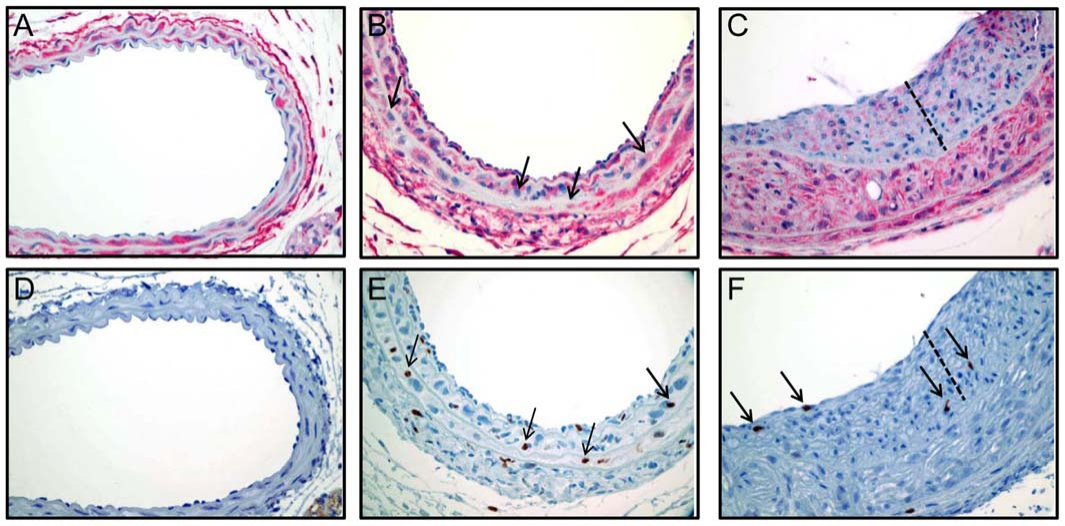
Vascular injury-induced Ki-67 and decrease in AKAP12 expression. Uninjured right carotid artery (panels A, D) or partial ligation of left carotid artery one week (panels B, E) and three weeks (panels C, F) post-injury were stained for either AKAP12 (red stain in panels A-C) or Ki-67 (brown stain in panels D–F). Arrows point to cells showing clear positivity for Ki-67 and reduced AKAP12. The dotted line in panels C and F represent the full thickness of the neointima. Note the marked decrease in AKAP12 staining after three weeks of the partial ligation injury. Original magnifications were 600×.

**Figure 7:**
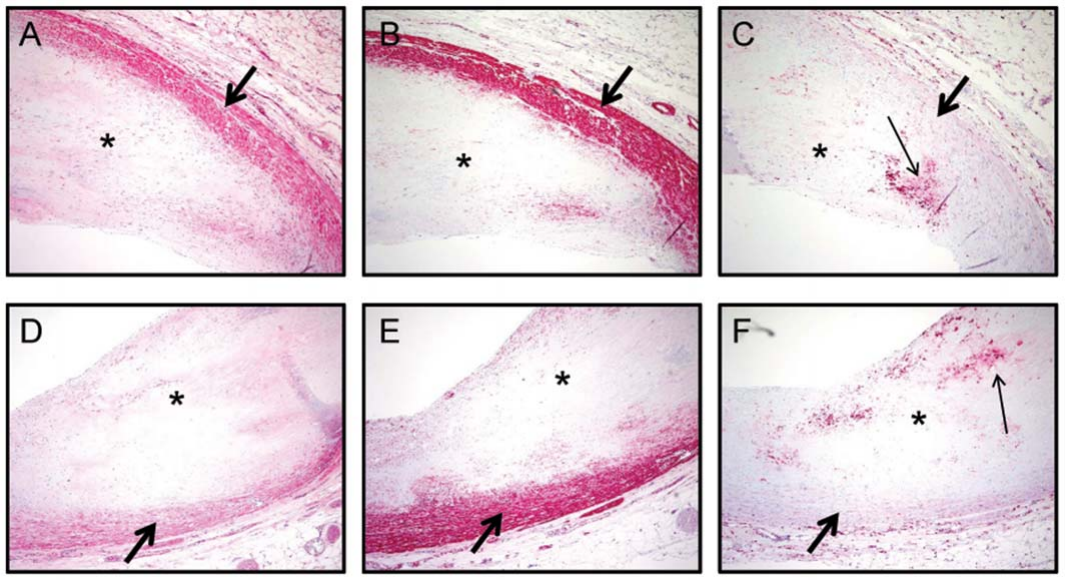
AKAP12 expression is reduced in human neointimal tissue. Serial sections of two independent atherosclerotic coronary vessels (panels A–C and D–F) stained for AKAP12 (panels A, D), CNN1 (panels B, E) and Ham56 (panels C, F). The red stain reveals positive immunoreactivity confined largely to the tunica media (AKAP12 and CNN1 indicated with a large arrow) or neointima (Ham56, indicated with small arrow). The neointima is labeled with an asterisk in each panel. Similar results have been found in multiple independent coronary arteries of varying atherosclerotic severity. Magnifications are 200×.

A hallmark of a tumor suppressor gene is reduced expression within tissues associated with accelerated growth. To examine expression of AKAP12 in vascular lesions associated with SMC growth, we used a mouse model of neointimal formation [33] combined with Ki-67 staining. IgG control stained vessels revealed no background staining (Figure S4). Uninjured carotid arteries exhibit abundant AKAP12 and no cell proliferation consistent with the contractile phenotype (Figure 6A, 6D and Figure S4). When such vessels are subject to a partial ligation injury [33], Ki-67 positive cells increase in the media and neointima of 1 week (20.87% Ki-67 positive cells, Figure 6E) and 3 week (4.5% Ki-67 positive cells, Figure 6F) vessels. In general, and consistent with data in the cancer field [34], Ki-67 positive cells showed weak AKAP12 staining, especially in 3 week injured vessels where a prominent neointima is manifest (Figure 6E, 6F). We also noted dramatic reductions in AKAP12 staining in the vessel wall following complete ligation of the carotid artery (Figure S4). Importantly, human atherosclerotic lesions showed virtually no AKAP12 expression within the neointima (Figure 7A, 7D). Such low AKAP12 expression correlates with reductions in CNN1 (Figure 7B, 7E), a SMC differentiation marker known to be reduced in atherosclerosis [35]. Similar findings have been seen in multiple independent atheromas of varying severity (data not shown). To rule out an intrinsic loss in immunoreactivity within the neointima of these vessels as an explanation for the loss in AKAP12 and CNN1 expression, adjacent sections were analyzed for macrophage content using the Ham56 antibody (Figure 7C, 7F). These results showed immunoreactive macrophages within the neointima indicating that loss in AKAP12 and CNN1 within the neointima is not a consequence of some intrinsic defect in immunolocalization. Thus, AKAP12 expression is attenuated in both experimental and human conditions of neointimal formation and such decreases appear to correlate with elevated SMC proliferation.

## Discussion

Over 50 AKAPs have been defined in the human genome. AKAP functions include the ability to compartmentalize multi-protein complexes in order to specify unique spatio-temporal signaling events involving PKA and other signaling moieties [31]. Here, we show that the natural retinoid, atRA, and several synthetic retinoids elicit rapid and robust induction of a specific isoform of AKAP12 (AKAP12β) in vascular SMC. The increase in AKAP12β is also seen in intact vascular tissue exposed to therapeutic levels of atRA. Although we identified a conserved DR2-RARE located in the proximal *Akap12β* promoter, transient and stable transfection studies revealed only weak (∼2-fold) activation of the *Akap12β* RARE with retinoid stimulation. Nevertheless, actinomycin D and ChIP assays suggest that retinoid signaling converges at the *Akap12β* promoter to effect gene transcription. A sensitive assay that detects pan-AKAP interactions with the PKA regulatory subunit II alpha indicates that AKAP12 is the only AKAP in SMC exhibiting dramatic increases in expression with retinoid treatment. Directed AKAP12β expression resulted in enhanced PKA activities and attenuated SMC growth. Finally, mouse and human vascular occlusive diseases were associated with reduced AKAP12 expression. Together, these studies establish AKAP12β as a strategic target of retinoid signaling thus providing a framework for further evaluation of this retinoid-AKAP12 axis in vascular SMC growth control and perhaps other cell types responsive to retinoid signaling.

AKAP12 (aka SSeCKS and Gravin) has demonstrable growth suppressive properties and is down-regulated in a variety of human neoplasms supporting the idea that AKAP12 is a bonafide tumor suppressor gene [32], [34], [36]–[40]. However, there is little information on the role of AKAP12 in non-neoplastic cell growth inhibition. Here, we demonstrate that the AKAP12β isoform reduces human and rodent vascular SMC growth and that such growth inhibition correlates with elevated PKA activity. Previous studies have demonstrated PKA-mediated signaling in the inhibition of vascular SMC growth as well as neointimal formation [41]–[43]. The precise signaling mechanisms underlying PKA-mediated SMC growth inhibition are unknown but likely relate to PKA redistribution within cells via AKAPs such as AKAP12. In this regard, we and others have demonstrated context-dependent and isoform-specific localization of AKAP12 within cells [29], [44], [45]. Interestingly, results shown in this report suggest that retinoid-induced AKAP12 concentrates in the peri-nuclear region in close proximity to PKA (Figure 4B). It is possible that such peri-nuclear localization of PKA may direct nuclear events such as the phosphorylation of CREB, which is known in some contexts to mediate SMC growth inhibition [46]. Further work is necessary to determine whether retinoid signaling via AKAP12 exerts effects on transcription factors, such as CREB, that may mediate SMC growth suppression. In this context, it will be informative to interrogate CREB factor binding, genome wide, following atRA stimulation of SMC to identify potentially important downstream target genes that may mediate AKAP12-dependent SMC growth inhibition.

Tumor suppressors are thought to normally effect growth inhibition. Although several tumor suppressor genes have been shown to be retinoid-inducible [47], none exhibit the rapid onset and high level induction observed with AKAP12β. It will therefore be informative to test whether treating neoplastic cells with natural or synthetic retinoids induces AKAP12β to counter-balance the documented silencing of the AKAP12α isoform [39], [40].

The *Akap12* locus comprises three independent promoters controlling unique isoforms with distinct intracellular localizations [29]. Such diversity in *Akap12* isoform expression exemplifies the complexity of the human genome with a total gene count only mildly greater than that in simpler vertebrate species. Limited information exists with respect to differential control of each *Akap12* promoter with virtually all work reporting effects on the proximal, *Akap12α* promoter. Initial studies demonstrated the importance of serum response factor-binding CArG boxes in the regulation of *Akap12α* promoter activity [48]. Adjacent GC-rich sequences in the *Akap12α* promoter were shown to undergo hypermethylation and gene silencing in various cancer cell lines; in contrast, the *Akap12β* promoter exhibited less gene silencing in cancer cells [34], [39], [40]. A subsequent study demonstrated a methylation-independent mechanism for *Akap12α* promoter silencing through the recruitment of HDAC1 [49]. More recently, the anti-proliferative agent dexamethasone was shown to weakly (2-fold) activate the *Akap12α* promoter without influencing *Akap12β* promoter activity, though no glucocorticoid responsive elements were reported [37]. We provide evidence here for a conserved RARE in the upstream *Akap12β* promoter region that is weakly responsive to retinoids. The DR-2 RARE in the *Akap12β* promoter is very similar to that of the known retinoid-response gene *Ca2* [30], which similarly exhibits weak activation with atRA stimulation (Figure 3B). Although we could not show consistent binding of a retinoic acid receptor to the RARE containing region, several lines of evidence suggest that retinoid receptors target this or perhaps a more distal *Akap12β* promoter region. First, actinomycin D experiments showed that the induction of *Akap12β* by atRA proceeds in a RNA polymerase II-dependent manner. Second, three synthetic retinoids that directly bind and activate retinoid receptors each induced *Akap12β*mRNA expression. Third, a ChIP assay revealed retinoid-mediated enrichment of acetylated histones in the region encompassing the *Akap12β* RARE. The weak activation of *Akap12β* promoter with retinoid treatment may imply the absence of key cofactor modifications needed to fully activate the promoter. Alternatively, there may exist remote regulatory elements, as reviewed elsewhere [50], controlling retinoid-mediated transcription of *Akap12β* from a distance. Methods in bacterial artificial chromosome transgenic mouse technology and related recombineering would be ideal approaches to address the latter possibility. Whatever the full extent of the mechanism(s) may be, this study clearly demonstrates that the increase in *Akap12* with retinoid treatment proceeds through *Akap12β*.

AKAP12 is not the only AKAP shown to suppress SMC growth responses. AKAP5 (aka AKAP79/AKAP75/AKAP150 in human, bovine, rat respectively) was previously demonstrated to inhibit SMC growth in vitro and, similar to our findings here, AKAP5 stimulated CREB-dependent transcriptional activity [51]. Moreover, local delivery of AKAP5 to the balloon-injured vessel wall reduced the extent of neointimal burden [51]. Since, as shown in this report, AKAP12 is reduced in human and rodent vascular lesions, one would not expect a compensatory, confounding influence of AKAP12 on AKAP5-mediated effects. In this context, there is similarity in key amino acid sequences between AKAP12 and AKAP5 [52]. Moreover, we have observed AKAP12 and AKAP5 share similar flanking genes suggesting that these two AKAPs are paralogs related via a segmental chromosomal duplication (Figure S5). Despite the functional and genomic similarities between AKAP12 and AKAP5, only AKAP12β is induced with retinoids as we were unable to show similar induction of AKAP5 (data not shown).

In summary, the results of this report document retinoid-induced stimulation of a specific AKAP12 isoform which exhibits growth suppressive properties, most likely via PKA-mediated signaling. Future studies should evaluate the extent of neointimal formation and the effects of retinoids in suppressing such growth in mice where the *Akap12* gene is genetically deleted [26]. Finally, effects of retinoids on AKAP12β expression should be evaluated in other disease contexts where cell proliferation is manifest (*e.g*., cancer).

## Materials and Methods

### Treatment of cells or animals with retinoids

Rat pulmonary artery SMC (PAC1) were grown and maintained as described previously [53]. Primary-derived rat aortic SMC (RASMC) were obtained from adult thoracic aorta of male Sprague-Dawley rats as described [54], grown in Dulbecco's modified Eagles Medium supplemented with 10% fetal bovine serum (FBS), and used between passage number 10–20. Human coronary artery SMC (hCASMC) were obtained from Cascade Biologics (Portland, OR) and grown in commercially-supplied growth medium per manufacturer's specifications. In all SMC cultures, we routinely validate their phenotype with a panel of SMC-restricted markers, including the SMC-restricted myocardin transcription factor [55],[56]. For retinoid stimulation, cells were synchronized for 24 hr in 0.25% FBS and then stimulated in fresh medium containing 0.25% FBS for the indicated times with 2×10^−6^ M of atRA, 13-cis RA, or 1 µM of one of three synthetic retinoids (AM80, BMY-46561, RAR agonists and BMS-188649, an RXR agonist). Control cells received 0.1% dimethylsulfoxide (diluent for retinoids) in medium containing 0.25% FBS. For in vivo experiments, we introduced either 10 mg/kg atRA in corn oil or an equivalent volume (∼50 µl) of corn oil alone by oral gavage to adult male FVB/N mice (n = 4 per treatment) and collected blood by intra-cardiac puncture 6 hr later for the determination of serum levels of atRA and its two major stereoisomers (9-cis RA and 13-cis RA) by HPLC as described previously [11]. The same mice were euthanized 18 hr later and aortas (stripped of endothelium and adventitia) harvested for total RNA isolation (as above) and RT-PCR with isoform-specific primers as described [29].

### cDNA cloning, Northern blotting, and RT-PCR

A rat-specific *Akap12* probe common to all three *Akap12* isoforms was PCR amplified from PAC1 cDNA using the following specific primers containing a 5 bp clamp and restriction sites (underlined): forward 5′- gatacggatccccaggatggggaagctga -3′ and reverse, 5′- gatacaagcttttccttgctctcttcttgg -3′ amplifying a 323 bp fragment of *Akap12*. A human-specific *AKAP12β* probe was PCR amplified from human coronary artery SMC (HCASMC) cDNA using the following specific primers: forward, 5′- gattaggatccccgctgaccactcacagag -3′ and reverse, 5′- gattgaagctttgtgatggtgatggtcccc -3′ amplifying a 421 bp probe. Rat *Akap12* isoform specific probes were PCR amplified as follows: for *Akap12α* (forward 5′- gataggtcgacgggagtagaagagccactg -3′ and reverse, 5′- cactcaagctttcaacgacttcttcctcc -3′ amplifying a 281 bp probe from GenBank Accession number AY695056); for *Akap12β* (forward 5′- gtatgtctagaatgctctgaggatagttagg -3 and reverse, 5′- ctatgaagcttctgtccaactgtgatggta -3′ amplifying a 156 bp probe from GenBank Accession number AY695057); and for *Akap12γ* (forward 5′- gataggtcgacaggcttggtagtttcgaagg -3′ and reverse, 5′- gatacaagctttctcgctgtccaagggaag -3′ amplifying a 115 bp probe from GenBank Accession number AY695058). Total RNA was isolated from control and retinoid-stimulated cells using TRIzol and fractionated in a 1.2% agarose gel, blotted to nitrocellulose, hybridized with ^32^P-dCTP-labeled probes, washed and exposed to Kodak X-ray film as described [21]. Alternatively, total RNA from retinoid-treated PAC1 SMC was analyzed by quantitative RT-PCR with BioRad's MyiQ icycler with primers (designed with Beacon Software, BioRad) specific to human *AKAP12β* (forward 5′- ttggcaggcaggagactagg -3′ and reverse, 5′ - tcgtgaacaaccgctgacttag -3′ amplifying a 187 bp product).

### Adenoviral construction

Full-length *Akap12β* cDNA with a carboxy-terminal FLAG epitope and LacZ were each cloned into pENTR/D-TOPO (Invitrogen) and then recombined into pAd/plDEST (Invitrogen) using LR clonase to create the Ad-AKAP12β or Ad-LacZ adenoviral constructs as described previously [48]. Viral production and titering were also done as described [48].

### Western blotting and R2 overlay assays

PAC1 SMC were synchronized with 0.25% FBS for 24 hr before stimulation with either 2×10^−6^ M atRA or 0.1% DMSO and protein extracted for Western blotting as described [48]. Primary antibodies to AKAP12 [22] (1∶2000 dilution), smooth muscle calponin (hCP, Sigma; 1∶10,000 dilution) or alpha tubulin (T5168, Sigma; 1∶2000) were applied to membranes followed by species-appropriate HRP-conjugated secondary antibodies. To evaluate the interaction of AKAP12 with the regulatory subunit of PKA, PAC1 SMC were stimulated with 2×10^−6^ M atRA or 0.1% DMSO for 12 and 24 hr and 75 µg total protein resolved in a large format 5% SDS-PAGE gel. The gel was then electro-blotted to a PVDF (Immobilon-P) membrane and dried for a radiolabeled RII overlay as described [57].

### Immunofluorescence microscopy

Cells were grown on glass chamber slides (Labtek), treated for retinoid stimulation as above, and at the indicated times, fixed, processed, and visualized with a fluorescence microscope as previously described [58]. Primary antibodies used were polyclonal rabbit anti-AKAP12 [22] and mouse anti-PKA RII alpha (BD Transduction Laboratories, Cat # 612242).

### Bioinformatics

Identification of a conserved RARE around the promoter of *Akap12β* isoform was performed using VISTA (http://www-gsd.lbl.gov/vista/index.shtml)(citation) by searching for pairs of consensus half sites (RGGTCA) and characterized variants situated within several nucleotides of each other that are conserved in sequence and relative position within a 20 kb window centered on the human, rat, and mouse *Akap12β* promoters [4].

### Cell transfections and reporter assay

To test and compare the *Akap12β* RARE for functionality, oligonucleotide primers comprising 3x multimerized RAREs from *Akap12β, Rarβ*, or the retinoid-response gene, carbonic anhydrase (*Ca2*) were inserted upstream of the minimal thymidine kinase promoter of tk-luciferase. PAC-1 SMC were transfected with the indicated construct, made quiescent for 24 hours using 0.25% FBS-containing DMEM, and subsequently stimulated with 2×10^−6^ M atRA for 24 hours. Dual luciferase assays were performed as previously described [29]. Data shown are representative of at least three independent experiments. Error bars represent the standard deviation from the mean. Data were analyzed by one-way ANOVA and Tukey's post-hoc test using GraphPad Prism software. A probability value less than 0.05 was considered statistically significant. In some experiments, similar luciferase assays were done in PAC1 SMC using a CREB reporter plasmid (PathDetect System, Stratagene) transfected with either an empty control vector or one carrying the AKAP12β open reading frame.

### Cell transduction and growth assay

Stable cell lines expressing doxycycline-inducible, Myc-tagged AKAP12β were generated using the T-Rex System (Invitrogen) in PAC1 SMC according to the manufacturer's specifications. To induce expression of AKAP12β, cells were treated every other day with 1 µg/ml doxycycline (Sigma) and levels of AKAP12β measured with a Myc antibody. HCASMC were grown to subconfluence in 6-well dishes and transduced with 300 infectious units per cell (ifu/cell) of either CMV-driven LacZ (Ad-LacZ) or CMV-driven AKAP12β (Ad-AKAP12β) in 2% FBS as described [48]. Following overnight culture in 2% FBS, culture medium was changed and replaced with 0.25% FBS for 24 hr to synchronize the cells. Cells were then stimulated with full growth medium and the number of trypan blue negative cells manually counted with a hemocytometer. At least three independent measures per time point were made in two independent experiments. Results are presented as the average of three replicates from one experiment ± the standard error of the mean.

### Ligation injury model

Male C57BL/B6 mice (30 g) were subject to partial ligation of the left carotid artery [33] and FVB/N mice were injured by complete ligation of the common carotid artery [59]. One and three weeks after injury, animals were perfusion fixed with neutral buffered formalin, vessels removed and processed, and sections (5 µm) of injured arteries distal to the ligature stained by immunohistochemistry with a polyclonal antibody to AKAP12 [22] or an antibody to Ki-67 to detect proliferating cells of the vessel wall. All animal studies were approved by the University of Rochester's Institutional Animal Care and Use Committee.

### Immunohistochemistry

Mouse vessels were fixed in 4% buffered paraformaldehyde and paraffin embedded. Samples of human coronary vessels with variable degrees of atherosclerosis were obtained from archived tissues in the University of Rochester Medical Center's Pathology Department. All tissues were sectioned at 5 micron thickness and slides were deparaffinized and rehydrated to PBS (pH, 7.4). Endogenous peroxidase activity was quenched using 3% aqueous hydrogen peroxide for 10 minutes and antigen retrieval was performed (for CNN1) utilizing heat induced epitope retrieval in 0.05% citraconic anhydride as described [60]. Primary antibodies (and their dilution) were as follows: rabbit polyclonal anti-AKAP12 (1∶500), anti-CNN1 (DAKO; 1∶1000), Ham56 (DAKO; 1∶1000), and Ki-67 (DAKO, 1∶100). Appropriate secondary biotinylated antibodies (Vector BA-2000, BA-1000, or DAKO rabbit anti-rat for Ki-67) were applied for 30 minutes at room temperature following washes in TBST. Immunoreactive signals were revealed by a 30 minute dark exposure to either alkaline phosphatase (Vector AK-5000) followed by Vector Red (Vector SK-5100) chromagen or horseradish peroxidase (Vector PK-6100) followed by diaminobenzidine chromagen. Specific immunoreactive product was indicated by inclusion of control isotype-matched IgG on adjacent sections that were handled in exactly the same manner as those stained with primary antibodies.

## Supporting Information

Figure S1 **A**, PAC1 SMC were treated with 1 µM AM80 for the indicated times or DMSO diluent and *Akap12β* mRNA measured by qRT-PCR (n = 3). **B**, PAC1 SMC were treated with 1 µM AM80 for 6 hr and immediately thereafter were exposed to 1 µg/ml actinomycin D or equal amount of water for the indicated times and *Akap12β* mRNA measured by qRT-PCR as in panel A. *Akap12β* mRNA was normalized to internal control *Gapdh* with the control (DMSO or 0 h) ratio set to a value of 1.
(TIF)

Figure S2 Extracts of PAC1 SMC were treated with DMSO or the synthetic retinoid, AM80 (1 µM), for 24 hrs and total protein analyzed for AKAP12, ACTA2, CNN1 and TUBA1 (control) proteins.
(TIF)

Figure S3 **A**, PAC1 SMC were co-transfected with a CREB reporter and either an empty vector or an AKAP12β expression plasmid to assess CREB activity in a luciferase assay. Results are expressed as normalized luciferase (see Methods). **B**, PAC1 SMC were co-transfected with EGFP (control) ± AKAP12β and phosphorylation of VASP assessed by immunoblotting.
(TIF)

Figure S4 Non-immunogenic IgG control antisera was applied to uninjured right carotid (A), 7 day injured left carotid (B), and 21 day injured left carotid (C). Panels D-F represent AKAP12 staining of uninjured carotid (D) and femoral (E) artery or a 7 day complete ligation injured carotid artery (F). Note loss of AKAP12 staining in the media of the injured vessel (F) as compared to normal vessels (D,E).
(TIF)

Figure S5 Schematic shows evidence of segmental chromosomal duplication with percent amino acid homologies between AKAP5 and AKAP12 and their paralogous flanking genes.
(TIF)
