# Supplementary figures and images for: Retinoid-Induced Expression and Activity of an Immediate Early Tumor Suppressor Gene in Vascular Smooth Muscle Cells
Source: PLoS One. 2011 Apr 5;6(4):e18538. doi: 10.1371/journal.pone.0018538 (PMC3071728; doi:10.1371/journal.pone.0018538)

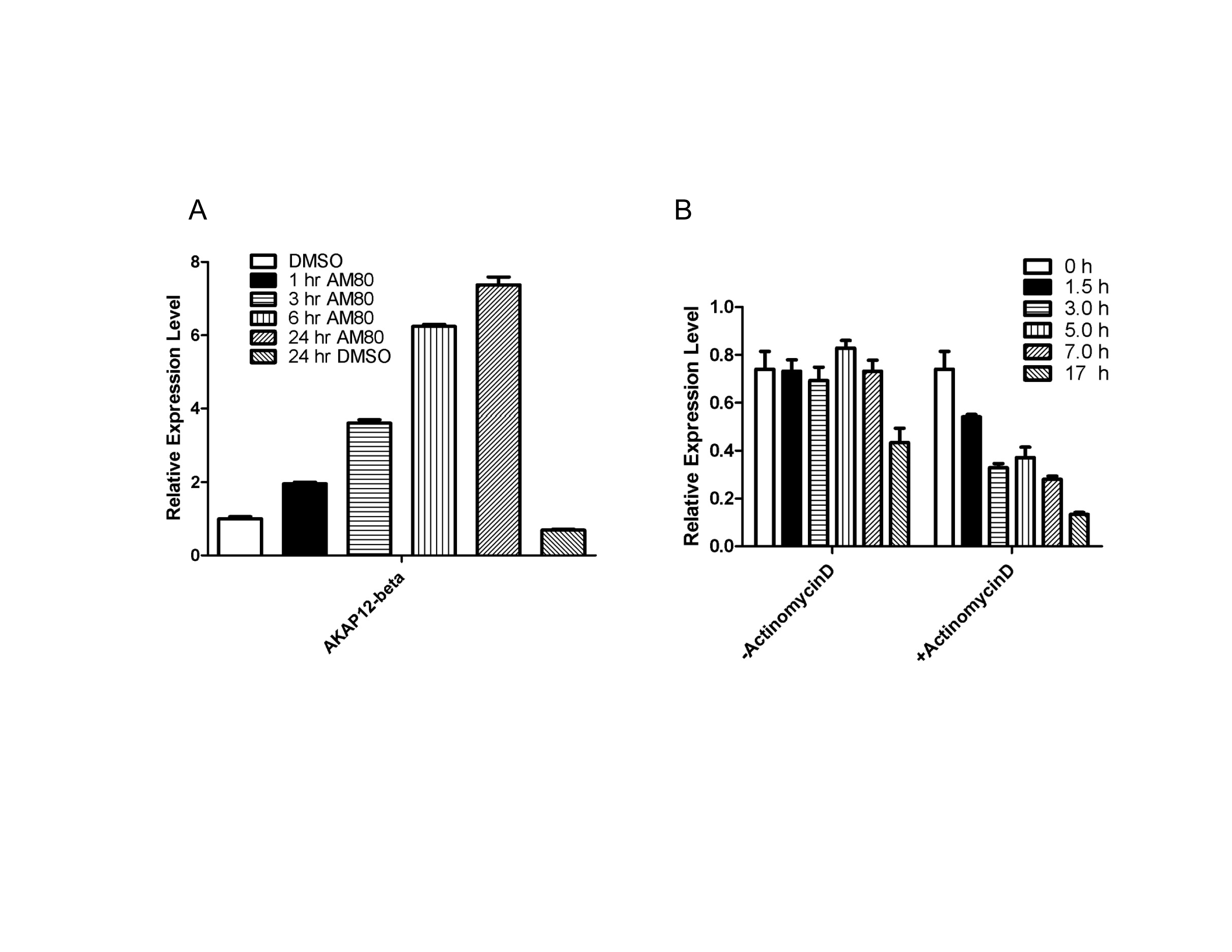

Supplement: Figure S1 — A, PAC1 SMC were treated with 1 µM AM80 for the indicated times or DMSO diluent and Akap12β mRNA measured by qRT-PCR (n = 3). B, PAC1 SMC were treated with 1 µM AM80 for 6 hr and immediately thereafter were exposed to 1 µg/ml actinomycin D or equal amount of water for the indicated times and Akap12β mRNA measured by qRT-PCR as in panel A. Akap12β mRNA was normalized to internal control Gapdh with the control (DMSO or 0 h) ratio set to a value of 1. (TIF) [file pone.0018538.s001.tif]

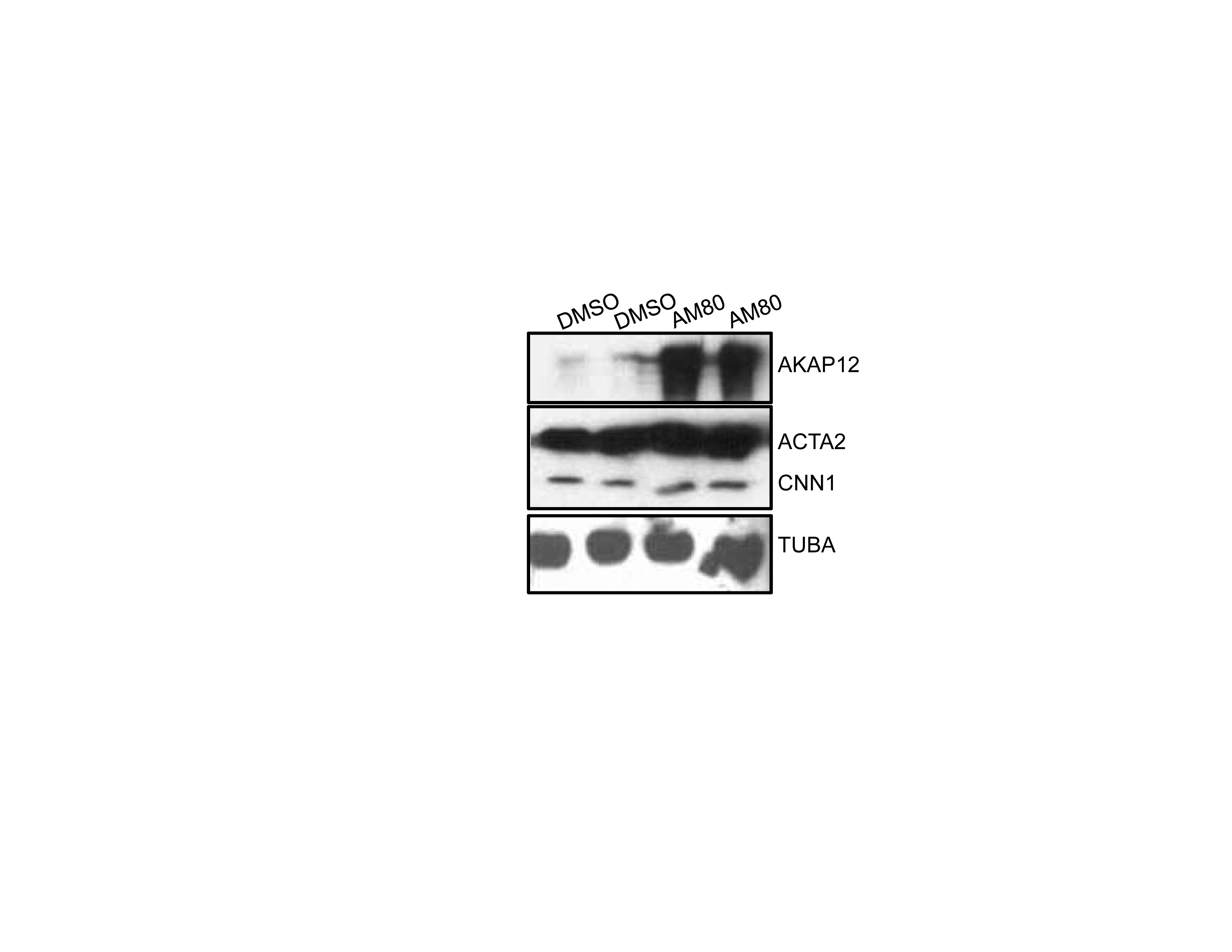

Supplement: Figure S2 — Extracts of PAC1 SMC were treated with DMSO or the synthetic retinoid, AM80 (1 µM), for 24 hrs and total protein analyzed for AKAP12, ACTA2, CNN1 and TUBA1 (control) proteins. (TIF) [file pone.0018538.s002.tif]

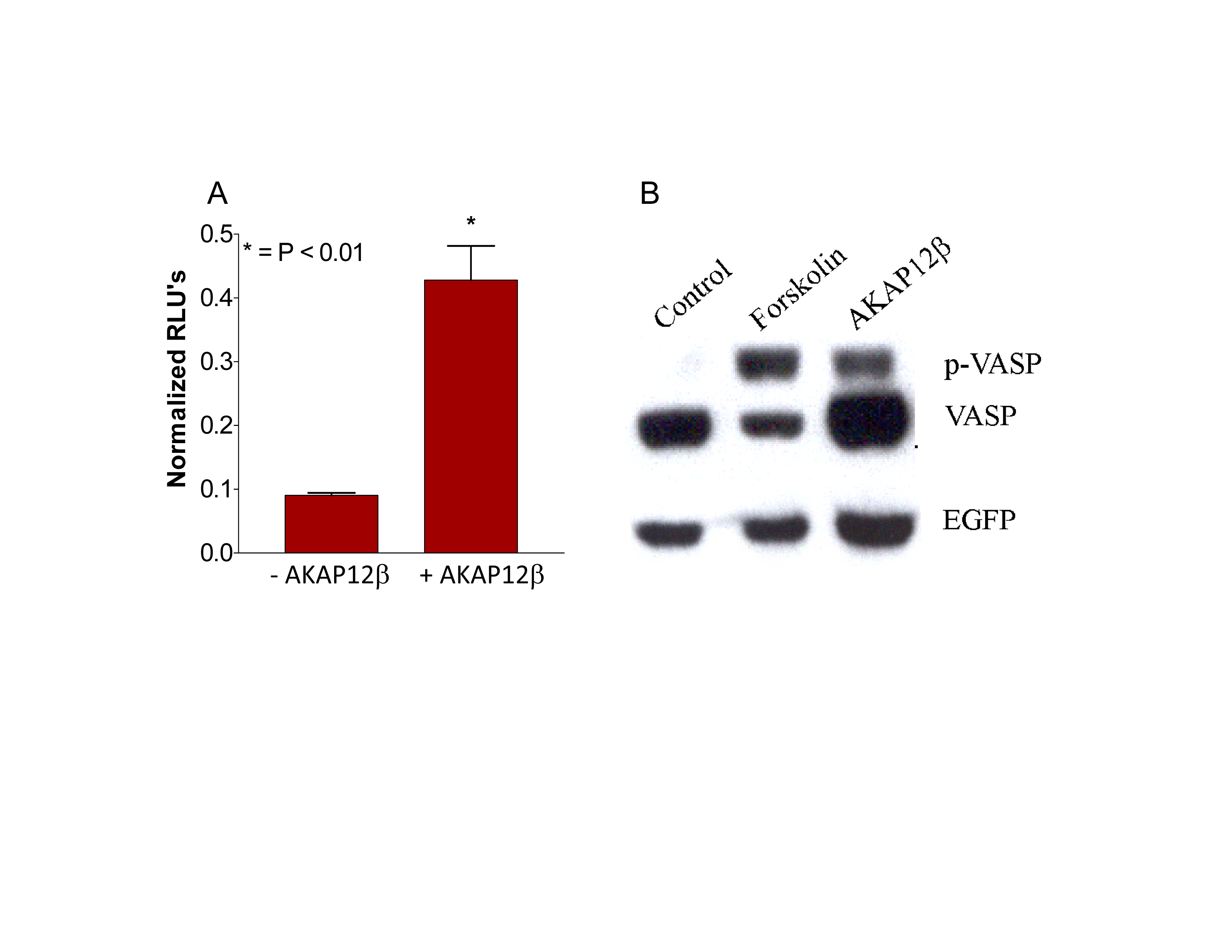

Supplement: Figure S3 — A, PAC1 SMC were co-transfected with a CREB reporter and either an empty vector or an AKAP12β expression plasmid to assess CREB activity in a luciferase assay. Results are expressed as normalized luciferase (see Methods). B, PAC1 SMC were co-transfected with EGFP (control) ± AKAP12β and phosphorylation of VASP assessed by immunoblotting. (TIF) [file pone.0018538.s003.tif]

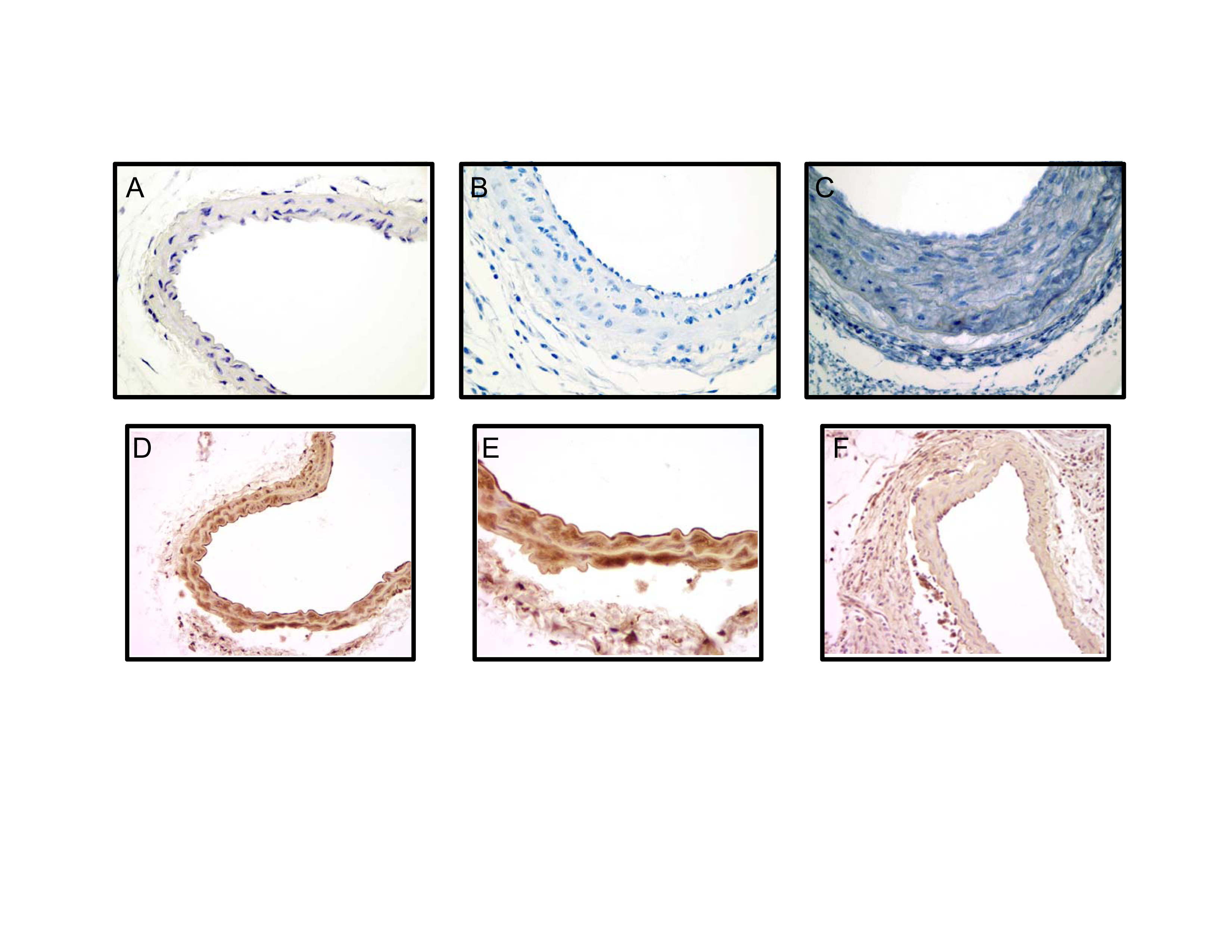

Supplement: Figure S4 — Non-immunogenic IgG control antisera was applied to uninjured right carotid (A), 7 day injured left carotid (B), and 21 day injured left carotid (C). Panels D-F represent AKAP12 staining of uninjured carotid (D) and femoral (E) artery or a 7 day complete ligation injured carotid artery (F). Note loss of AKAP12 staining in the media of the injured vessel (F) as compared to normal vessels (D,E). (TIF) [file pone.0018538.s004.tif]

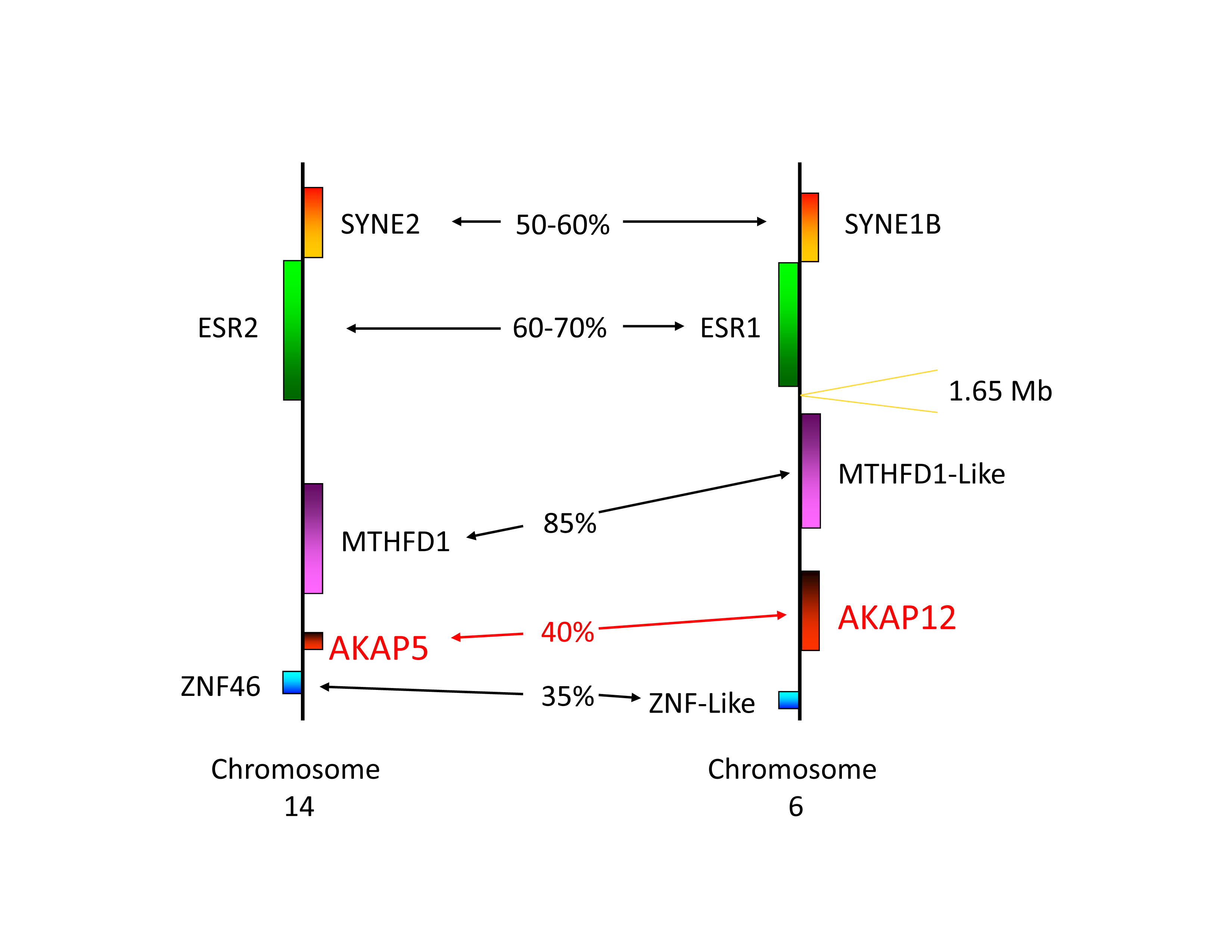

Supplement: Figure S5 — Schematic shows evidence of segmental chromosomal duplication with percent amino acid homologies between AKAP5 and AKAP12 and their paralogous flanking genes. (TIF) [file pone.0018538.s005.tif]
